# A transcription factor for cold sensation!

**DOI:** 10.1186/1744-8069-1-11

**Published:** 2005-03-22

**Authors:** Susan J Kim, Zhican Qu, Jeffrey Milbrandt, Min Zhuo

**Affiliations:** 1 Department of Physiology, University of Toronto, Medical Sciences Bldg, Rm 3342, 1 King's College Circle, Toronto, ON M5S 1A8, Canada; 2 Washington University, Departments of Pathology and Psychiatry, St. Louis, MO 63110, USA

## Abstract

The ability to feel hot and cold is critical for animals and human beings to survive in the natural environment. Unlike other sensations, the physiology of cold sensation is mostly unknown. In the present study, we use genetically modified mice that do not express nerve growth factor-inducible B (NGFIB) to investigate the possible role of NGFIB in cold sensation. We found that genetic deletion of NGFIB selectively affected behavioral responses to cold stimuli while behavioral responses to noxious heat or mechanical stimuli were normal. Furthermore, behavioral responses remained reduced or blocked in NGFIB knockout mice even after repetitive application of cold stimuli. Our results provide strong evidence that the first transcription factor NGFIB determines the ability of animals to respond to cold stimulation.

## 

Cold sensation is an important physiological protective function. The sensation of feeling cold is conducted by cold-sensitive ion channels that are expressed in primary sensory fibers [1,2]. Nerve growth factor (NGF) is required for the differentiation and survival of primary sensory fibers [3]. Nerve growth factor-inducible B (NGFIB) (also known as Nur77) is a transcription factor that is rapidly induced by NGF [4]. However, little is known if NGFIB regulates the function of primary sensory fibers. Here, we use genetically modified mice that do not express NGFIB to show the essential requirement of NGFIB for cold sensation in adult mice.

To determine if deletion of NGFIB affects sensory responses, we first measured behavioral responses to acute noxious stimuli in wild-type (WT) and NGFIB knockout mice (NGFIB^-/-^). No significant difference in tail-flick response latencies was observed (WT, n = 9 mice; NGFIB^-/-^, n = 15 mice, Fig. 1a), suggesting that spinal thermal nociceptive transmission was not significantly altered in NGFIB^-/- ^mice. Similarly, no difference was found in response latencies to the hot-plate test (55°C), another behavioral test that requires both spinal and supraspinal processes of nociceptive information (WT, n = 9 mice; NGFIB^-/-^, n = 15, Fig. 1b). Therefore, behavioral responses to acute noxious thermal stimuli were indistinguishable between NGFIB^-/- ^and WT mice. To further examine if changes in thermal responses are temperature dependent, we performed the hot-plate test at five different temperatures (from 44 to 52°C). We found no significant difference between WT (n = 6 mice) and NGFIB^-/- ^mice (n = 6 mice; Fig, 1c). One possibility for the roles of NGFIB in sensory transmission is that it may depend on sensory modality. To test this hypothesis, we evaluated behavioral responses of WT and NGFIB^-/- ^mice in another two behavioral tests using mechanical and cold stimuli. For mechanical stimuli, we evaluated the mechanical threshold to Von-Frey filaments applied to the tail. As shown in Fig. 1d, we found no significant difference in tail withdrawal thresholds (WT, n = 6 mice; NGFIB^-/- ^mice, n = 12 mice). Next, to explore possible behavioral changes to cold stimulus, we used a cold-plate test [5] in both WT and NGFIB^-/- ^mice. We placed mice on the cold-plate and NGFIB^-/- ^mice showed significantly reduced escaping responses (n = 12 mice, Fig. 1e). By contrast, WT littermates (n = 6 mice) showed behavioral nociceptive responses (Fig. 1a), as was previously reported in C57/6J wild-type mice [5]. Finally, we wanted to examine if repetitive exposure to cold stimuli causes behavioral responses to cold in NGFIB^-/- ^mice. In a previous report [5], repetitive measurements of mice in the cold-plate test caused sensitization of behavioral responses. Similarly, we found that WT mice (n = 6 mice) showed significantly sensitive responses on the cold-plate (Fig. 1f). However, in NGFIB^-/-^, no sensitization (i.e., significant reduction of escape latency) was found, indicating a permanent loss of cold sensation (n = 12 mice, Fig. 1f). To test if the loss of cold response is selective for NGFIB, we also examined behavioral responses to cold in NGFIA knockout mice (NGFIA^-/-^). We found that mice lacking NGFIA showed normal responses to cold (n = 6 mice, Fig. 1e).

**Figure 1:**
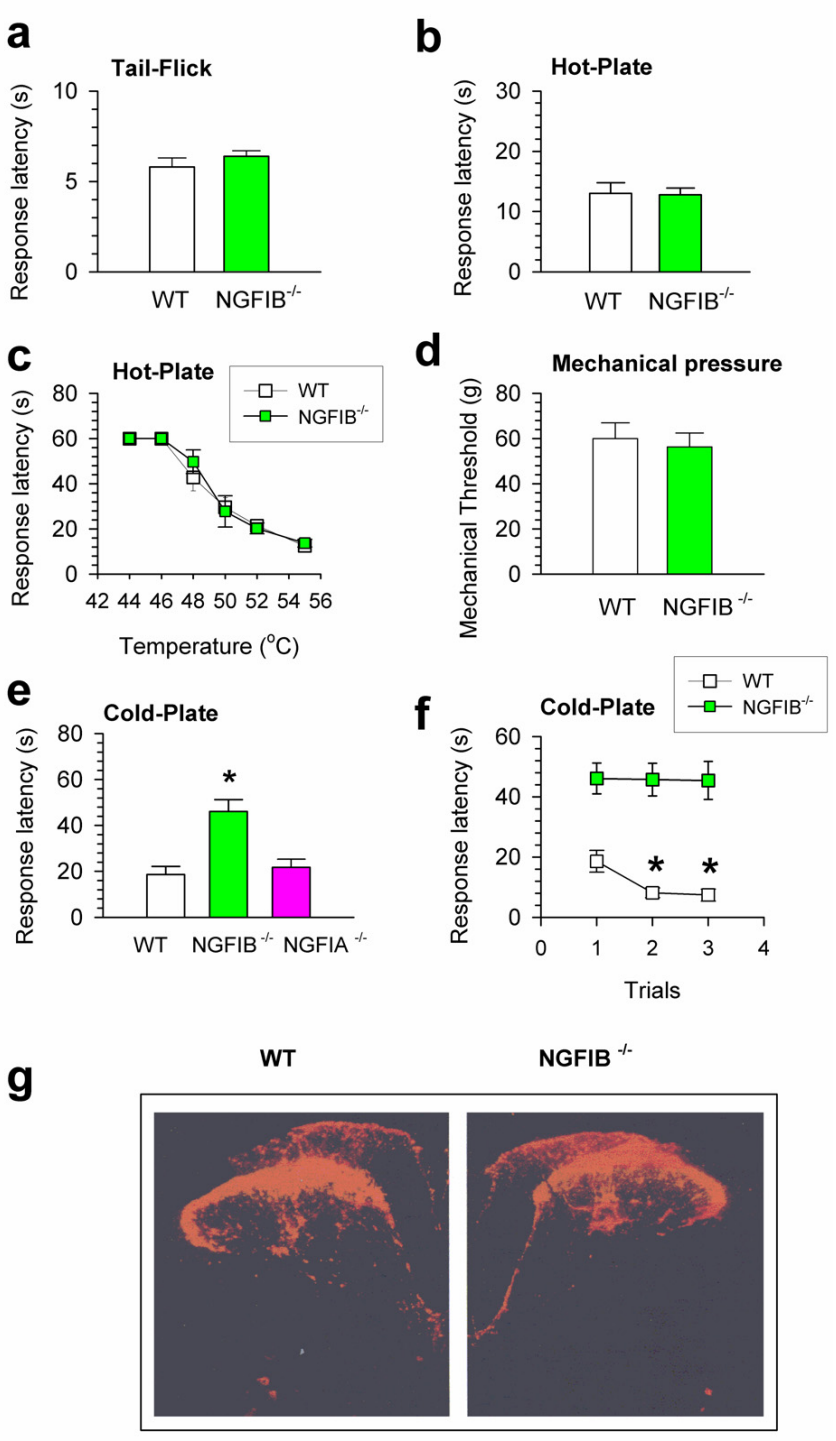
**Selective genetic abolishment of cold sensation in NGFIB knockout mice**. **a-b**. Behavioral responses of wild-type littermates (WT) and NGFIB knockout (NGFIB^-/-^) mice in the spinal tail-flick test (a) and the hot-plate test (b). **c**. Behavioral responses of WT and NGFIB^-/- ^mice in the hot-plate test with different plate temperatures. No significant difference was detected between two groups. **d**. Behavioral responses of WT and NGFIB^-/- ^mice to mechanical pressure applied to that tail. **e**. Behavioral responses of WT, NGFIB^-/- ^and NGFIA^-/- ^mice on the cold-plate (0°C). Response latencies of NGFIB^-/- ^mice were significantly reduced as compared with WT littermates or NGFIA^-/- ^mice. * P < 0.05 as compared with WT littermates or NGFIA^-/- ^mice. **f**. Behavioral responses in the cold-plate test after repetitive measurements performed at 5 min intervals. While WT mice showed significant sensitization (i.e., significant reduction in response latencies), NGFIB^-/- ^mice showed not sensitization. *. P < 0.05 compared with the first behavioral response latency. **g**. Immunostainings of CGRP-positive labels in the spinal cord lumbar dorsal horn of WT and NGFIB^-/- ^mice.

Thus, behavioral results demonstrate that defective cold responses are rather selective in NGFIB^-/- ^mice. Considering the recent report of cold receptor ANKTM1 expression in CGRP positive DRG cells [6], we wanted to examine if CGRP containing sensory terminals are affected in NGFIB^-/- ^mice. As shown in Fig. 1g, we found a similar distribution of CGRP positive staining in the spinal dorsal horn of WT and NGFIB knockout mice. Our results strongly suggest a role for NGFIB in behavioral responses to noxious cold responses, and the behavioral defect is unlikely due to the loss of CGRP-positive fibers in the spinal cord. This finding indicates a selective role of NGFIB in sensory responses to cold stimuli but not heat stimuli, a selective physiological function for a rather general transcription factor. Future studies are needed to address if the loss of cold sensation in NGFIB-/- mice is due to dysfunction of cold receptors and/or the inhibition of cold receptor expression. Considering recent progress in identifying proteins/channels for encoding cold receptors, we believe that NGFIB will offer a novel transcription factor for possible regulation of these cold-related receptors.

## Materials and methods

Adult male mice (wild-type and mutant NGFIB or NGFIA mice) were used. NGFIB^-/- ^and NGFIA^-/- ^were generated by homologous recombination, using a targeting vector which contained a neomycin resistance gene insertion in the region recoding the amino-terminal domain of NGFIB. Wild-type and homozygous mutant NGFIB or NGFIA mice were obtained by crossing heterozygous mutant mice bearing a targeted mutation of the NGFIB gene. Genotypes were identified by PCR analysis of genomic DNA extracted from mouse ear tissue. Mice were maintained on a C57BL/6 background and were age-matched in each experiment. Both wild-type and mutant mice were well-groomed and showed no signs of abnormality or any obvious motor defects. No indication of tremor, seizure or ataxia was observed. As it was impossible to visually distinguish mutant mice from wild-type mice, experimenters were blind to the genotype. The Animal Studies Committee at Washington University and University of Toronto approved the experimental protocols.

The spinal tail-flick reflex was evoked by focused, radiant heat applied to the underside of the tail. The latency to reflexive removal of the tail away from the heat was measured by a photocell timer. In the hot-plate test, mice were placed on a thermally-controlled metal plate (Columbia Instruments; Columbus, Ohio). The time between placements of a mouse on the plate and licking or lifting of a hind paw was measured with a digital timer. Mice were removed from the hot plate immediately after the first response. For the cold-plate test, mice were placed on the iced surface maintained at 0°C. The time between placements of a mouse on the plate and licking or lifting of a hind paw was measured with a digital timer. For immunohistochemical staining, spinal cord sections were prepared on a cryostat and incubated with the CGRP antibody.

Results were expressed as mean ± s.e.m. Statistical comparisons were made with one- or two-way analysis of variance (ANOVA) with the *post-hoc *Scheffe F-test for immunocytochemical experiments, or the Student-Newmann-Keuls test for behavioral experiments, to identify significant differences. In all cases, *p *< 0.05 was considered statistically significant.
